# Feasibility of Fiber Reinforcement Within Magnetically Actuated Soft Continuum Robots

**DOI:** 10.3389/frobt.2021.715662

**Published:** 2021-07-08

**Authors:** Peter Lloyd, Zaneta Koszowska, Michele Di Lecce, Onaizah Onaizah, James H. Chandler, Pietro Valdastri

**Affiliations:** Science and Technology of Robots in Medicine (STORM) Laboratory, School of Electronics and Electrical Engineering, University of Leeds, Leeds, United Kingdom

**Keywords:** magnetic continuum manipulators, soft robotics, continuum robots, magnetic actuation, soft surgical robots, magnetic soft continuum robots, fiber reinforced soft robots

## Abstract

Soft continuum manipulators have the potential to replace traditional surgical catheters; offering greater dexterity with access to previously unfeasible locations for a wide range of interventions including neurological and cardiovascular. Magnetically actuated catheters are of particular interest due to their potential for miniaturization and remote control. Challenges around the operation of these catheters exist however, and one of these occurs when the angle between the actuating field and the local magnetization vector of the catheter exceeds 90°. In this arrangement, deformation generated by the resultant magnetic moment acts to increase magnetic torque, leading to potential instability. This phenomenon can cause unpredictable responses to actuation, particularly for soft, flexible materials. When coupled with the inherent challenges of sensing and localization inside living tissue, this behavior represents a barrier to progress. In this feasibility study we propose and investigate the use of helical fiber reinforcement within magnetically actuated soft continuum manipulators. Using numerical simulation to explore the design space, we optimize fiber parameters to enhance the ratio of torsional to bending stiffness. Through bespoke fabrication of an optimized helix design we validate a single, prototypical two-segment, 40 mm × 6 mm continuum manipulator demonstrating a reduction of 67% in unwanted twisting under actuation.

## 1 Introduction

Elastomeric soft continuum manipulators (CMs) represent a promising and highly active research area among the soft robotics community Burgner-Kahrs et al. (2015). A major subset of these robots are magnetically actuated, tip driven CMs Edelmann et al. (2017), Kim et al. (2019) which offer the potential to fulfill and enhance the role of the surgical catheter. Magnetic CM’s are popular due to their extrinsic actuation, uniquely offering near limitless potential for miniaturization da Veiga et al. (2020), Bira et al. (2020). The concept of a shape forming soft magnetic robot was introduced for an untethered application in Diller et al. (2014). This was developed into a tethered “cilium-like” manipulator in Lum et al. (2016) and further specialized to catheter-like shapes in Lloyd et al. (2020a).

The appeal of the shape forming CM (over tip-driven approaches) lies in the higher dexterity of motion, offering a potentially dramatic reduction in uncomfortable and sometimes damaging anatomical contact forces during navigation within the body. This is of particular relevance for applications which require large bending deformation such as gastrointestinal and endovascular interventions da Veiga et al. (2020). Full shape-forming can allow for better lumen-following and thus a reduction in friction; permitting the use of softer materials. In order to achieve minimal contact navigation, the lengthwise magnetization profile of the CM and the applied actuating fields must be specifically configured.

**FIGURE 1:**
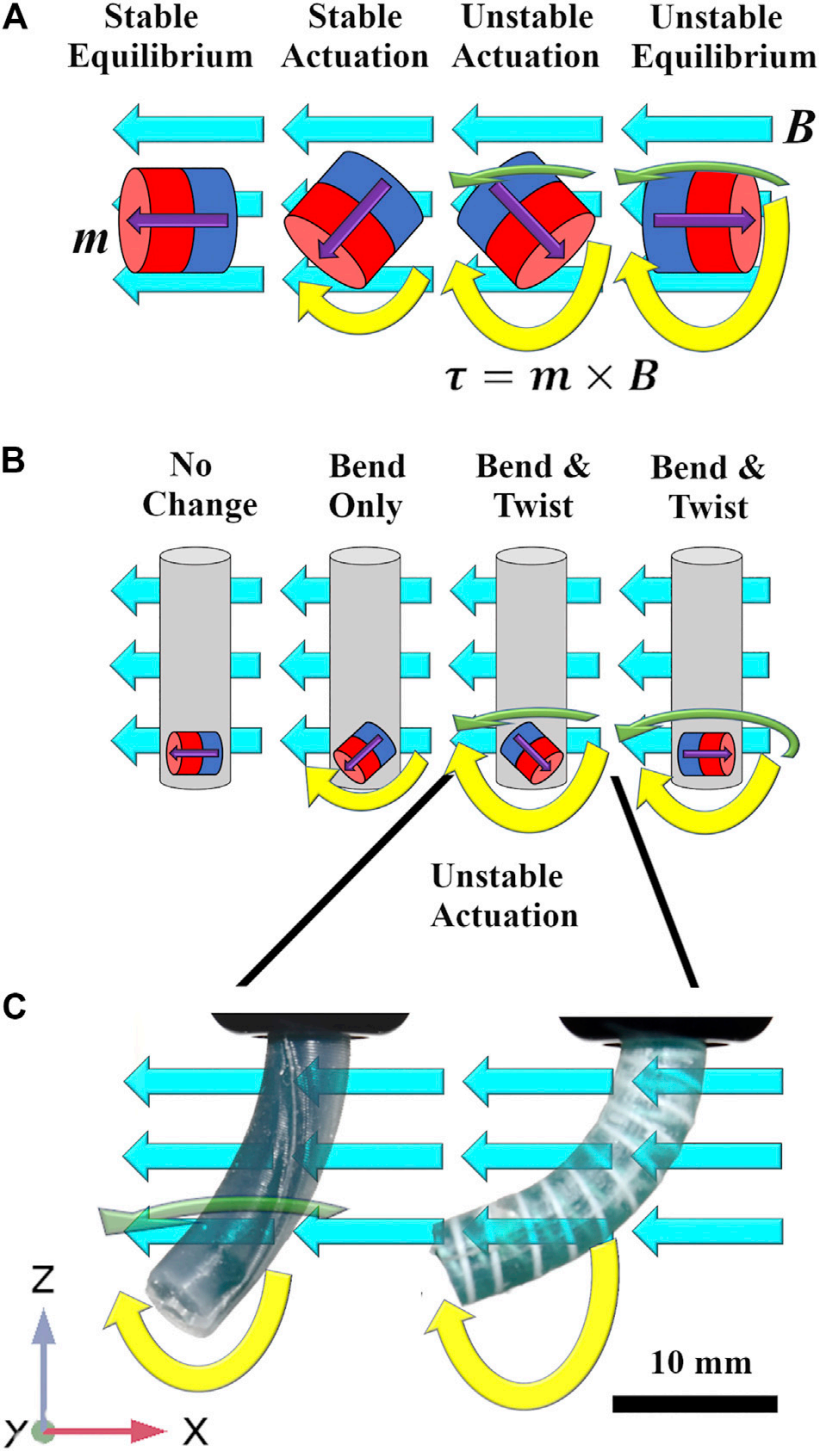
**(A)** Magnets in free space subject to a homogeneous actuating field [$-B_{x}$ 0 0] (turquoise arrows) and with magnetization [$m_{x}$ 0 $m_{z}$] (purple arrows). As actuation lies in the x-z plane, theoretically, torque acts only about the *y* axis (yellow arrows) and is zero in the unstable equilibrium position. In practice, due to the inverted pendulum instability, torque exists about both z (green arrows) and *y* axes. Unstable modes of actuation also produce magnetic torque which increases with deflection. **(B)** Once embedded in a CM these unstable torques translate as both bending and twisting deformations. **(C)** Photographs from experimental actuation; the unreinforced sample on the left succumbs to the twisting instability about the *z*-axis. The reinforced sample on the right constrains the unstable twisting mode of actuation resulting in larger magnetic torque in the desired, bending, mode of deflection.

**FIGURE 2:**
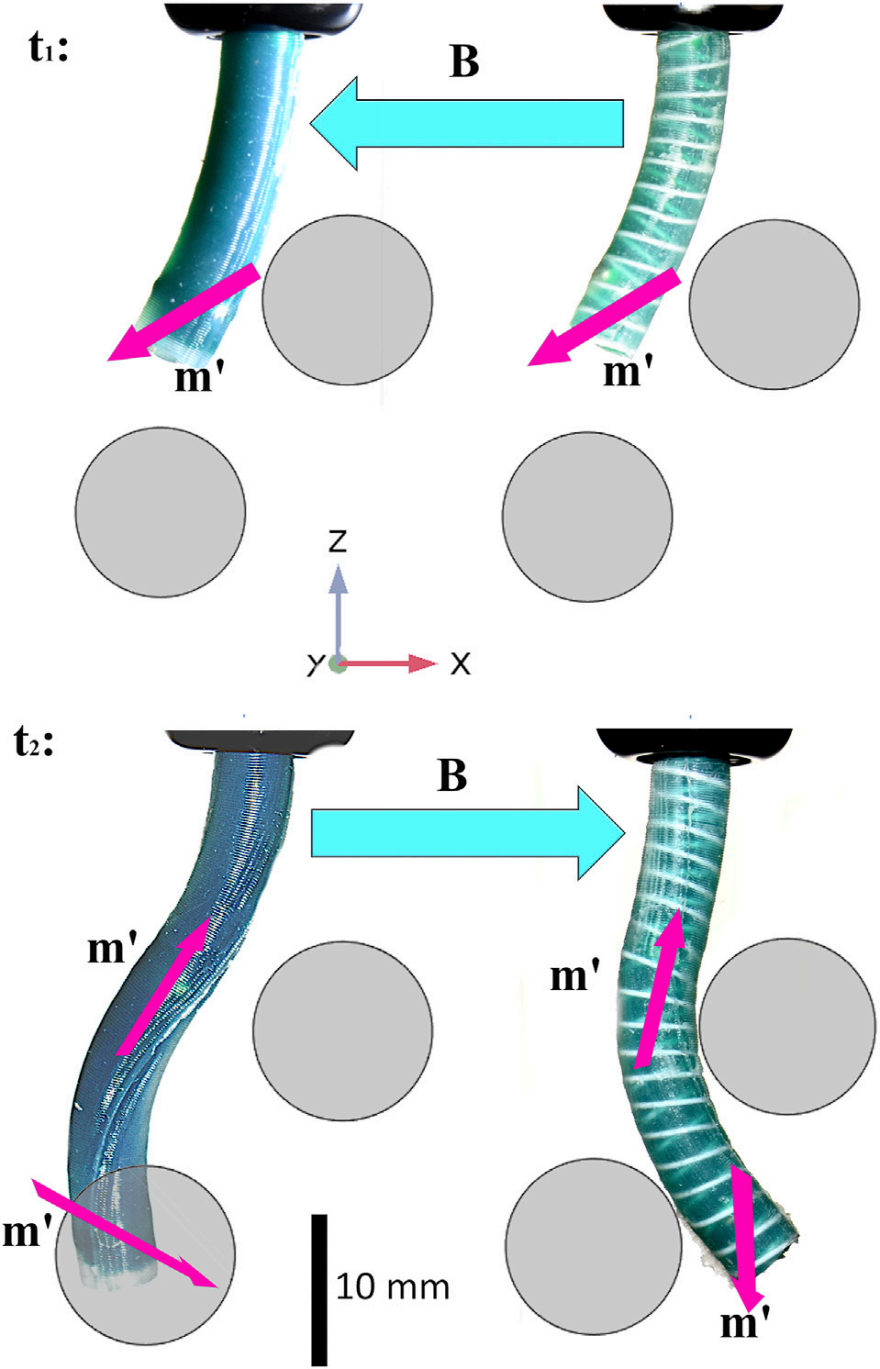
A minimal reproducible example of the impact of magnetic instability arising during manipulator navigation [as introduced in Lloyd et al. (2020b)]. Specific magnetization profiles and time variant actuating fields achieve follow-the-leader motion without imparting contact forces on the surrounding environment. Here, deformed magnetization vectors (**m′**) are shown as purple arrows and applied fields (**B**, which change with respect to time) as turquoise (referential magnetizations are omitted to avoid clutter but are shown in the supporting video and in Section 5). The range of magnetization vectors required to achieve desired profiles generates unavoidable instabilities where the angle between **m** and **B** is obtuse. As can be seen bottom left, the unreinforced manipulator is prone to twist about the, lower resistance, local *z*-axis causing a loss of desired deformation; this twist is also clearly shown in the Supplementary Video 1. If the manipulator was rotated $180^{\circ }$ about global z for the second step of the navigation, the twisting torque would disappear but the bending torque about the lower magnet in the deformed state would also diminish such that the desired manipulator shape could not be achieved. In the reinforced specimen this twisting is minimized and deformation is focused about the *y* axis thus achieving the required contact free navigation. In this example, obstacles are represented by 2D background images placed in order to illustrate the objective.

Key to the development of the shape forming magnetic CM is the ability to support unstable combinations of magnetization and applied field (illustrated in Figure 1 and demonstrated in the supporting video). A combination of diverse magnetic signatures Lum et al. (2016) and time-variant actuating fields Lloyd et al. (2020b) will inevitably create obtuse angles of actuation. Such obtuse angles will also unavoidably occur in the case that actuation is generated via external permanent magnets [e.g. Pittiglio et al. (2020)] which cannot be turned off and must transition from one field to another. These modes of actuation represent a promising option in terms of efficient generation of a clinically relevant workspace but unwanted twist in this situation could potentially disrupt a navigation to a dangerous degree. Large angles between the CM magnetization and the applied field produce an “inverted pendulum” instability. The CM will then inevitably twist about its long axis (*z*-axis in Figure 1) in a search for the minimum energy pose. An example of this behavior is shown in Figure 2. Theoretically, closed loop control strategies such as the adaptive dynamic control demonstrated in Barducci et al. (2019) could be employed to counteract this instability. However, this solution would prove highly impractical for real life applications due to the challenges of monitoring and sensing within the human body, particularly with reference to magnetically actuated tools Vrooijink et al. (2013), Norton et al. (2019). A further alternative to the eradication of twisting would be to incorporate axial rotation as a modeled and controlled degree of freedom. This option would add to the complexity of the robot mechanics and would also contribute some out-of-plane manipulability. For the purposes of this investigation we are interested in maximizing in-plane bending deformation and as such we attempt to focus magnetic torque into the bending primitive and not lose energy in twisting.

One potential solution for improving open-loop control of these soft robots is to produce an anisotropic elasticity distribution by reinforcing the elastomer with higher stiffness fibers in order to restrict torsion whilst still permitting bending. This approach has parallels with organically evolved systems such as the collagen fiber reinforcing of the earthworm and related invertebrates, frequently referenced by the soft robotics community Calderón et al. (2016). Following this concept, Kreig and Mohseni Krieg and Mohseni (2017) investigated high deformation anisotropic strain restriction and the geometric arrangement of fibers in elastomeric sheets. The commonly employed Mckibben artificial muscle Connolly et al. (2015) consists of a pneumatic bladder providing a uniform hydrostatic pressure and strain restricting fibers capable of producing a range of motion primitives Marchese et al. (2015), Connolly et al. (2017). More recently fluidic elastomer actuators have been developed and extensively exploited to offer a diverse range of pre-programmed mechanical responses to pneumatic pressure Polygerinos et al. (2015), Zhang et al. (2020). Strain restriction has proved highly effective for soft pneumatic actuators, however, this approach has yet to be applied within the field of magnetic CM research.

In this preliminary study we demonstrate the application of strain-limiting fiber reinforcement within magnetically actuated soft CMs for improved open-loop actuation stability. Magnetic CMs have their own specific challenges, notably, small-scale manufacturing, small actuation wrenches and unique input instabilities. Here we present a numerical analysis of the potential usefulness of fiber reinforcement which has previously never been proposed or explored. Our experimental work is for validation purposes only and significant development will be required particularly around fabrication and control before we can perform a full experimental analysis.

The specific application being considered is a multi-segment shape forming CM, or *tentacle*, with multiple magnetic elements as introduced in Lloyd et al. (2020a). Here we demonstrate using a single prototypical 2-segment design, as shown in the supporting video, which represents a subset of the full shape forming CM. The workflow through the paper is as follows: In Section 2 we set out our goals and research methods. In Section 3 we define the underlying theory behind the magnetically actuated elastomeric catheter and our associated numerical model. In Section 4 we present the results of our single segment optimization and in Section 5 we extend the study into a multi-segment design including fabrication and experimental evaluation. We conclude and discuss in Section 6.

## 2 Problem Definition

An algorithm for the selection of a magnetization profile and actuating fields for a fully shape forming catheter was demonstrated, in a virtual environment, in Lloyd et al. (2020b). The contribution of this work was to develop an optimization routine to derive specific magnetization profiles and time variant actuating fields to achieve follow-the-leader motion without imparting contact forces on the surrounding environment during navigation. One unavoidable outcome of this insertion process is a number of potentially unstable actuation arrangements. Referring to Figure 2, it can be observed in the second time step ($t_{2}$), the field associated with the lower of the two fixed magnets creates an obtuse angle with the actuating field. This condition *could* be specified as a parameter to be minimized (or excluded) in any optimization routine, however, the wide range of magnetization directions required to achieve any practically useful desired profile will inevitably generate such problems, particularly as the number of magnetized segments increases. The consequence of this instability is often catheter twist about the lower resistance *z* axis; this twisting can be observed in the unreinforced samples of both Figures 1, 2 and the supporting video. This twisting is highly unpredictable and thus extremely difficult to simulate.

The objective of our work is to minimize the twisting primitive whilst maximizing the bending primitive, thus allowing practicable shape forming under a wider range of magnetization-magnetic field conditions. There are many potential solutions to the general problem of twist minimization in CMs but we are constrained by certain application specific requirements. In order to preserve the softness and malleability of the manipulator, and given the very low wrenches involved, we must employ soft, flexible reinforcing material in a sparse manner to produce anisotropic response to actuation. Specifically, we are attempting to minimize helical strain whilst allowing axial strain using only soft material. As such, our proposed solution to this multivariate problem, as inspired by Connolly et al. (2015), Polygerinos et al. (2015), Connolly et al. (2017) and Krieg and Mohseni (2017) uses a double (and therefore symmetrical) helix of reinforcing fiber.

**FIGURE 3:**
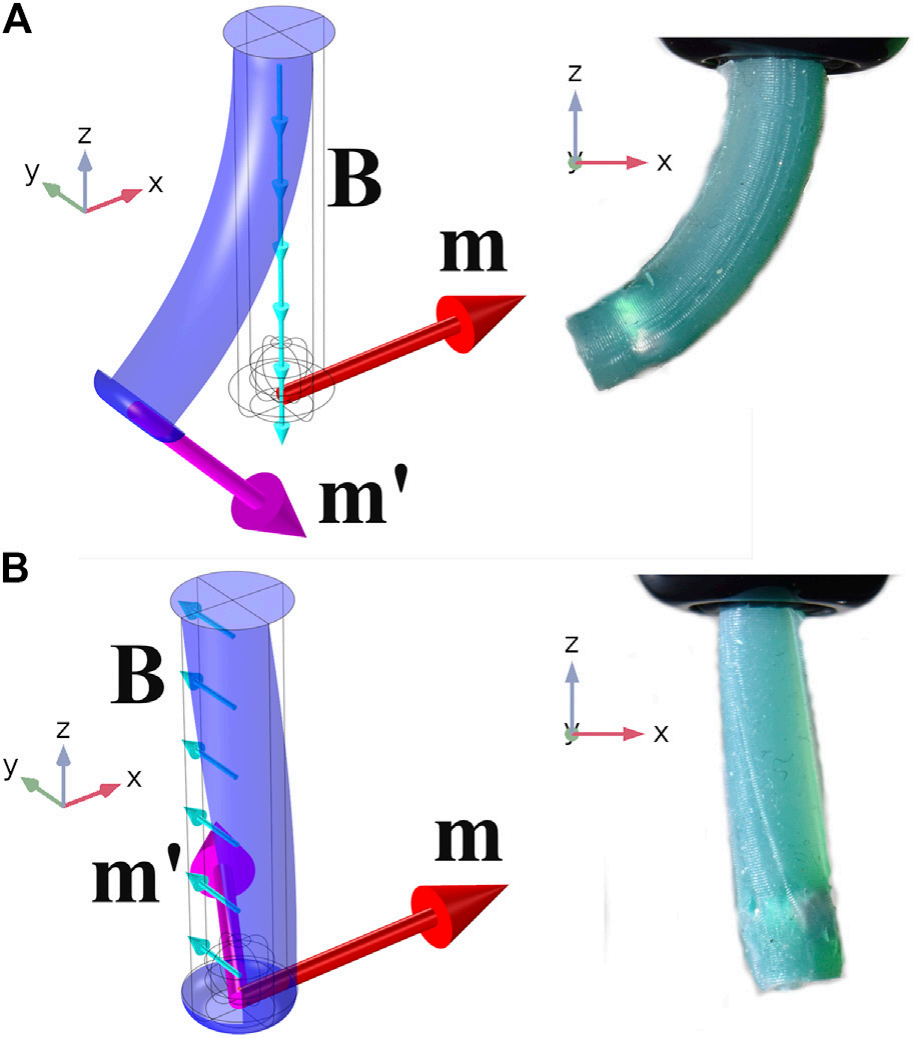
Pure bending about *y* **(A)** and pure twisting about *z* **(B)**. The unreinforced Single Segment demonstrating the two non-trivial motion primitives (Pure bending about x and y are characteristically identical). On the left are isometric views of numerical simulations and on the right, planar (x–z) images of unreinforced prototypes. Referential magnetization vectors (**m**) are shown as red arrows, deformed magnetization vectors (**m′**) as purple and applied fields (**B**) as turquoise. Simulations recreated experimental results for the range −20mT ⩽ B ⩽ 20mT with errors (mean ± standard deviation) of $3.6^{\circ }\pm 2.7^{\circ }$ demonstrating accuracy of the magnetic and material modeling assumptions.

To quantify prospective designs, we established baseline tests using a Finite Element (FE) simulation of a 20 mm long × 6 mm diameter elastomeric catheter as shown in Figure 3. A single 3.2 mm × 3.2 mm cylindrical permanent magnet (PM) is embedded at the tip (PM’s center 2 mm from the distal end) and the catheter is secured at its proximal end. The axis of magnetization of the PM was aligned in the positive *x* direction as indicated by the red arrows in Figure 3. The bending primitive was tested by applying a uniform magnetic field, **B**, in the negative *z* direction (Figure 3A). This actuating field generates a magnetic moment about the positive *y* axis and thus results in pure bend. The same sample, when subject to a **B** field in the positive *y* direction (Figure 3B), generates a magnetic moment about the positive *z* axis and thus results in pure twist. With these test cases we separate the two deformation primitives which, in most realistic states, will co-exist. We use this separation to assess the relative susceptibility of a range of designs to the two primitives before they are combined in experimental prototypes.

Evaluating designs using FE simulation allows exploration of a wide range of design properties. Here we explore variations in elastic modulus of elastomer $\left(E_{silicone}\right)$, filament diameter of reinforcing fibers (*d*), fiber angle $\left(\theta_{H}\right)$ and number of helices (*N*). The samples shown in Figure 3 are unreinforced and these results constitute the baseline, or N=0, result. We explore the design space with the underlying objective of determining an arrangement of fibers which constrain twisting without excessively constraining bending.

## 3 Modeling and Simulation

### 3.1 Material Model

We consider a soft catheter fabricated from elastomeric material (Ecoflex00-30, Smooth-On, United States) with strain limiting fibers made from Polylactic acid (PLA) (Material 1,613, Ultimaker, Netherlands), both with readily available material properties. The reinforcing fibers are assumed to be linearly elastic and are thus represented in the numerical model by the two constants; elastic modulus $E_{PLA}$ = 2.4 GPa and Poisson ratio *v* = 0.4. Due to the large elastic deformation experienced by the catheter we represent the silicone using an isotropic, strain energy based hyperelastic model. The commonly employed Yeoh model, which represents a trucation of the Rivlin power series to the third order for only the first principle stretch Yeoh (1993) and Nair (2009), is represented by:

$$W=\sum_{i=1}^{n}C_{i}{\left({\bar{I}}_{1}-3\right)}^{i}+\frac{1}{2}\kappa {\left(J-1\right)}^{2},$$ (1)

where *W* is Strain energy, *n* = 3, is the order of the model, *κ*, is bulk modulus, ${\bar{I}}_{1}$ is the first principle stretch (not to be confused with second moment of area) and *J* is the Jacobian determinant of the deformation gradient tensor the measure of compressibility. The choice of model and parameters were based on the work of Steck et al. (2019) who conclude that the Yeoh model with parameters $C_{1}$ = 17,000 Pa, $C_{2}$ = −200 Pa, $C_{3}$ = 23 Pa and *κ* = 300 kPa gives the most accurate representation of Ecoflex00-30 for strains up to 100%.

### 3.2 Magnetic Model

From Maxwell’s third equation, ∇.**B** = 0; divergence of a magnetic field must always be zero. From the fourth equation for a current-free field ∇× **B** = 0; curl must also be zero. From the Lorentz force, a magnetic dipole moment **m** will be pulled in the direction of any spatial gradient of **B** with a force **F** = (**m**.∇) **B**. That same magnetic dipole will also experience a resultant torque, linearly proportional to applied field strength τ = **m** × **B** (where **B**, **m**, **F**, $\tau \in {\mathbb{R}}^{3}$). Throughout this paper we assume homogeneity of applied fields such that ∇ **B** = 0 and thus **F** = 0. Consequentially, the only active component of the wrench is τ.

### 3.3 Balance of Momentum

The torque acting on the tentacle as a consequence of the interaction of the actuating magnetic field and the magnetization of the embedded PM is counteracted by gravity and by the elastic properties of the material. In the case of the unreinforced tentacle this material response is exclusively generated by the isotropic bulk elasticity of the silicone. However, for the reinforced tentacle we must also include the tensile and compressive stresses of the fibers. At all measured states the system was assumed quasi-static and as such the sum of all forces and the sum of all torques at all points must be zero.

A reduction in the ratio of bending to twisting stiffness is achieved by constraining the principle stretches of the twisting primitive using a material of far greater elastic modulus than the host elastomer $\left(E_{PLA}\approx 40E_{silicone}\right)$. These higher stiffness fibers have extremely high aspect ratios such that they can be assumed to only support compressive and tensile stresses (not shear stresses); the mechanical definition of a truss element. This characteristic, when oriented correctly, can be exploited to minimize the impact of any strain limitation in the bending primitive.

It is possible to characterize elastic moduli as a function of direction, to accommodate a difference between tensile and compressive stiffness. This characteristic is explored in some depth in Cappello et al. (2018) and would add significant complication to any analysis of fiber reinforcing. In the presented case we only consider reinforcing materials that can be considered to have a scalar elastic modulus.

### 3.4 Finite Element Method

The simulation environment was built in COMSOL mutiphysics v5.5 (COMSOL AB, Stockholm, Sweden) using the solid mechanics module to simulate the silicone elastomer and PM’s, and the truss module to simulate the fiber reinforcement.

NdFeB magnets have an elastic modulus of 150 GPa, ($\approx 10^{6}$ times the stiffness of Ecoflex 00-30) and can therefore be modeled as rigid domains of infinite stiffness. Magnetic torque is applied via mechanical moment on a rigid domain. This torque is a function of applied field and magnetization in each of the three directions but also of the three dimensional rotation of the rigid domain. This iterative calculation was hardcoded into the mechanical moment input, without loss of accuracy, circumventing the need to include direct consideration of electromagnetics with its attendant non-linearity and mesh requirements. Actuation was simulated as a 3.2 mm × 3.2 mm N52 NdFeB cylindrical PM embedded into the elastomer subject to a homogeneous magnetic flux density of −20 mT ⩽|B|⩽ 20 mT. The remanent magnetization of N52 NdFeB is given as $B_{r}$ = 1.455 T (K&J Magnetics, Inc., United States).

The material employed for the reinforcing fiber is, in the numerical model, represented as a chain of truss elements. These elements are attached to the solid mechanics module using a prescribed displacement option effectively imposing zero slippage between the fibers and the elastomer; a valid assumption when interactive shear forces are sufficiently low.

All models were meshed using tetrahedral elements, free-formed by the COMSOL auto-mesh generator. The larger, two segment, simulation required 26,000 tetrahedral finite elements and took 60 s to converge utilizing Newton-Raphson iterations within the MUltifrontal Massively Parallel sparse direct Solver (MUMPS) option. For a parametric sweep of applied actuating field |**B**| from −20 to +20 mT in 1 mT increments this represents a 40 min run-time on a 3.2 GHz, 32 GB, 16 core Intel Xeon Gold processor.

## 4 Single Segment Optimization

Here we isolate bending and twisting in our single segment numerical simulations with the objective of assessing the impact of variations in our design variables on these respective primitives. The optimized solution which we converge upon will then be implemented for a multi-segment tentacle in Section 5. The four design variables considered in this study are; fiber angle, $\theta_{H}$, number of helices, *N*, elastic modulus, *E* and filament diameter, *d*. The objective of the optimization is to increase twisting stiffness with as little increase in bending stiffness as possible. The first two design variables are considered in Section 4.1 and the latter two, which are inextricably linked, in Section 4.2.

### 4.1 Variation in Fiber Angle and Number of Helices

**FIGURE 4:**
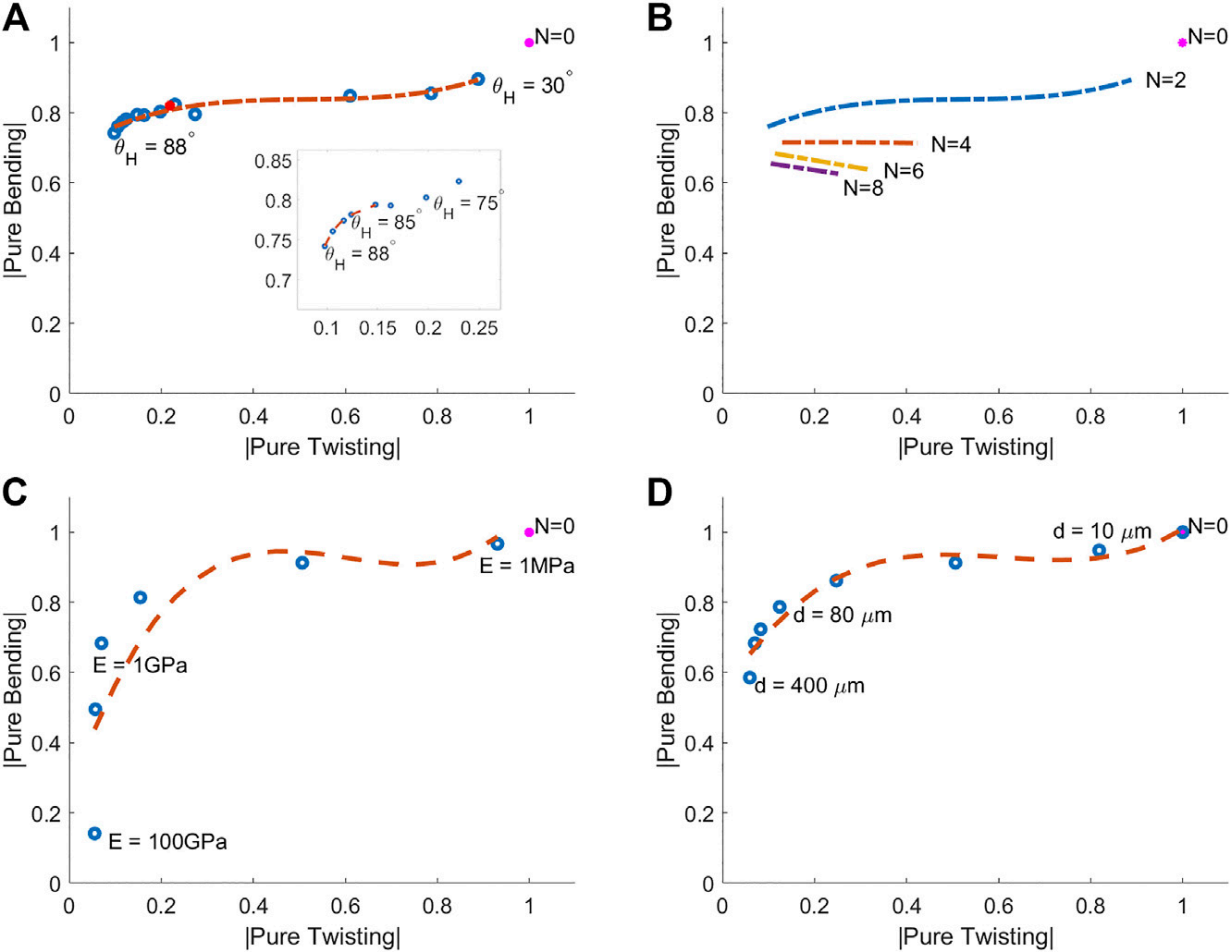
Pure bending vs. pure twisting for various helix arrangements under 10 mT actuation. **(A)** Variation in fiber angle ($\theta_{H}$). **(B)** Variation in number of helices (*N*). **(C)** Variation in elastic modulus (*E*), **(D)** Variation in diameter of reinforcing fibers (*d*). The unreinforced sample appears as *N* = 0 at the point (1,1). All twisting (*x*-axis) and bending (*y*-axis) results are shown as a proportion of this unreinforced result. The objective therefore is to move as far to the left on the *x*-axis as possible whilst staying as high as possible on the *y*-axis. **(A)** Shows the double helix (*N* = 2) arrangement for $30^{\circ }\;⩽\;\;\theta_{H}\;⩽\;\;88^{\circ }$ and the inset shows a zoom of the left hand extremity of the curve for higher $\theta_{H}$. The red spot represents the degenerate case $\theta_{H}=90^{\circ }$ which is included for completeness. This is not in fact a helix but reinforcing rings spaced at 1 mm centers along the tentacle length. The curve gradient equals unity at $\theta_{H}=85^{\circ }$. **(B)** Shows the impact of increasing number of helices at various fiber angles. Results shown are: *N* = 2 taken from **(A)**, *N* = 4, 6, 8, $\theta_{H}=50^{\circ }:85^{\circ }$. **(C)** Shows the effect of a logarithmic increase in elastic modulus for fixed filament diameter (*d* = 200 μm), fiber angle $\left(\theta_{H}=85^{\circ }\right)$ and number of helices (*N* = 2). The curve gradient equals unity at *E* = 1 GPa. **(D)** Shows the effect of increasing filament diameter for fixed elastic modulus (*E* = 1 GPa), fiber angle $\left(\theta_{H}=85^{\circ }\right)$ and number of helices (*N* = 2). The curve gradient equals unity at *d* = 200 μm.

For the purposes of this study fiber angle $\left(\theta_{H}\right)$ is defined as the angle between the fibers and the longitudinal axis of the manipulator (Krieg and Mohseni (2017)). Figure 4A shows the effect on bending and twisting of varying fiber angle for a double helix (N=2) of filament diameter d= 200 μm and modulus of elasticity E=1 GPa. An increase in fiber angle produces a higher ratio of bending to twisting only beginning to fall away above $\theta_{H}=85^{\circ }$.

A higher fiber angle increases the length of fiber per unit length of manipulator (as pitch is proportional to the tangent of the negative of fiber angle). An alternative method for increasing fiber length per unit length of the manipulator is through the use of additional concentric helices. To maintain symmetry, these helices must be increased in pairs (one left-handed helix and one right-handed helix). Figure 4B shows the effect of varying the number of helices for a variety of fiber angles (where filament diameter d= 200 μm and modulus of elasticity E=1 GPa). It is clear from Figure 4B that N=2 dominates alternative arrangements, this result also supports ease of manufacture.

### 4.2 Variation in Elastic Modulus and Filament Radius

The elastic and geometric characteristics of the fiber reinforcement, relative to the elastomer, are crucial to successful strain restriction. If the elastic modulus or fiber diameter are too high then bending flexibility will be lost and if too low then twisting will be unconstrained and instability will persist. There is, however, a fundamental inter-relationship between the two variables. The uni-axial stiffness of a truss member is a linear function of EA (where *A* is cross-sectional area) so any increase in *E* can be counteracted by a corresponding decrease in *A* and vice versa. The tipping point at which the ratio of bending to twisting stiffness starts to drop in both Figures 4C,D lies where the constant EA is approximately equal to 30 N.

In theory, there are infinite ways to produce an EA value of approximately 30 N, however, in practice limitations in material type and achievable dimensions exist. If a non-ferrous metal such as Titanium were used as reinforcing material $\left(E_{Titanium}=100\;\text{GPa}\right)$, a filament diameter of d= 20 μm would be required. This does not represent an easy-to-manufacture scale. Conversely, were we to manufacture our reinforcing filament from a highly flexible polymer such as Ninjaflex $\left(E_{Ninjaflex}=10\;\text{MPa}\right)$ we would need a filament diameter, d= 2 mm which would not physically fit into the catheter at our desired scale. The consequence of these practically imposed limits is that, to fulfill an optimal EA of 30 N, we need a mid-range stiffness material fabricated at a physically realistic diameter.

## 5 Shape Forming Tentacle

**FIGURE 5:**
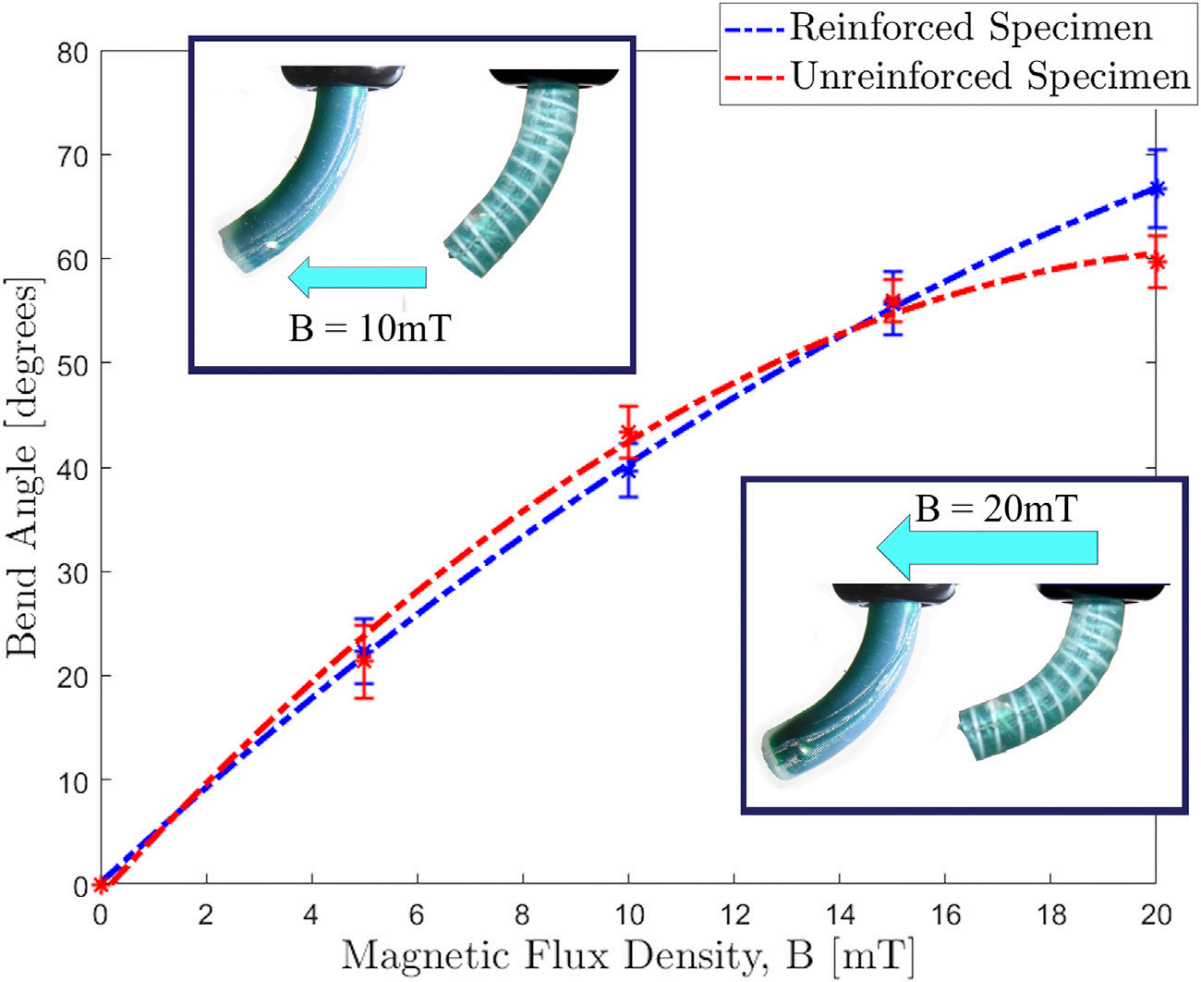
Actuating magnetic flux density (|**B**|) against bend angle (**θ**) for unreinforced and reinforced single segment specimens, magnetized in the unstable orientation ($135^{\circ }$ between **B** and **m**) as detailed in Figure 1. At lower actuating fields the absence of reinforcing fibers results in a softer CM and thus permits a higher level of deformation (as observed in the 10 mT insert). As applied field increases the reinforced specimen resists the unstable twisting primitive and thus generates greater magnetic torque about the desired axis, we therefore observe greater bending deformation (20 mT insert).

**FIGURE 6:**
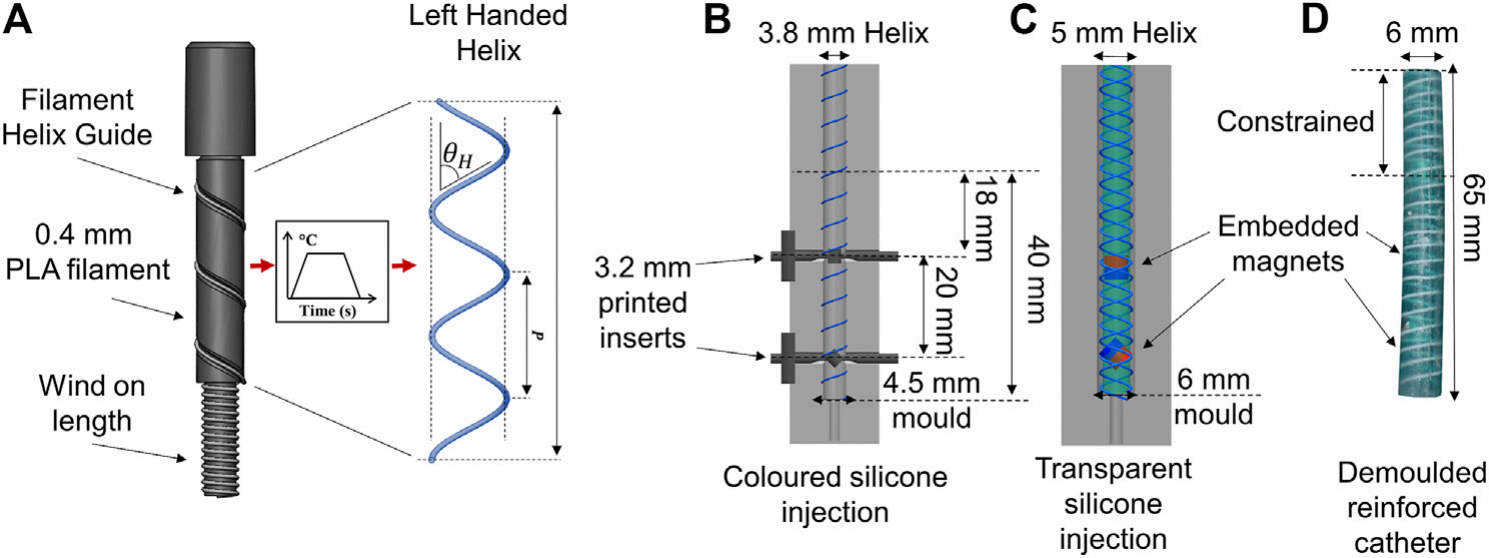
Fabrication process of the reinforced magnetic catheter. **(A)** Helix formation with a 0.4 mm PLA fiber at a fiber angle of $\theta_{H}$. Helix pitch (P) is the parallel length of one complete turn such that $tan\left(90^{\circ }-\theta_{H}\right)=P/\pi D$ (*D* = helix diameter). **(B)** Molding of tentacle core with inner helix and printed inserts to create magnet cavities, EcoFlex00-30 was injected into the mold and cured. **(C)** Magnets placed in cavities and outer, opposing helix secured around the tentacle core. EcoFlex00-30 injected into the mold and cured to create a 1.5 mm outer coating. **(D)** The demolded two segment tentacle featuring double helix reinforcing.

Figure 5 demonstrates, for an unstably actuated ($135^{\circ }$ between **B** and **m**) single-segment CM, the potential of fiber reinforcing to reduce twisting while allowing bending for obtuse magnetization-magnetic field angles. The use of reinforcement, however, is most beneficial in multi-segment cases where magnet directions are non-parallel Lloyd et al. (2020b). Accordingly, in this section we apply the concept to a two segment design. As shown in Figure 6, we have a CM of 40 mm total unconstrained length and 6 mm diameter with two 3.2 mm × 3.2 mm cylindrical N52 NdFeB PMs embedded at 18 and 38 mm centers from the mechanical constraint (z = 0). Both magnets are aligned such that their magnetization vectors lie in the x-z plane. The upper embedded magnet is aligned at a $90^{\circ }$ angle to the applied field and the lower at a $135^{\circ }$ angle.

### 5.1 Numerical Result

**FIGURE 7:**
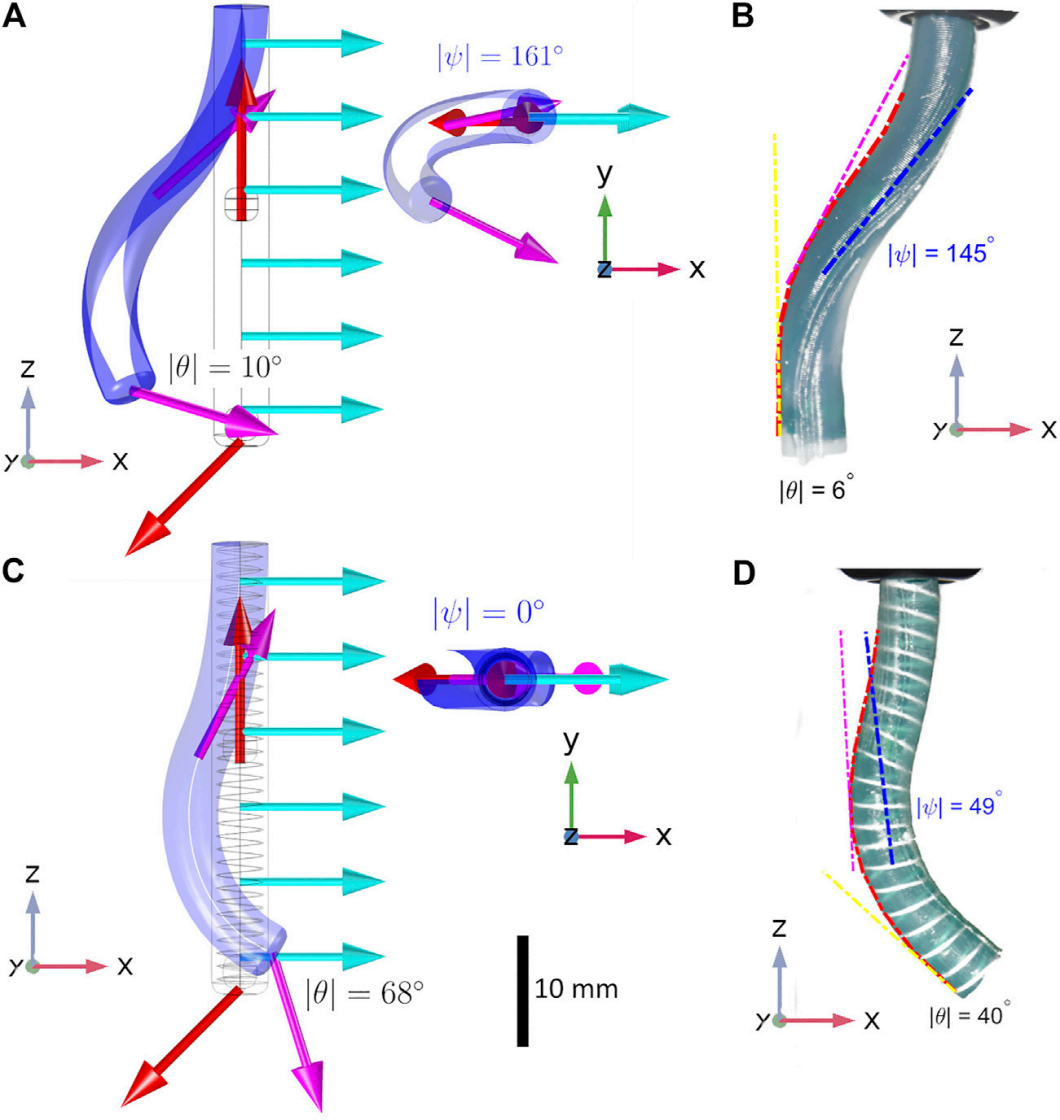
Both reinforced and unreinforced specimens have identical referential magnetizations (red arrows in the numerical simulation) and are subject to the same actuating field ($\text{B}={\left[2000\right]}^{T}$ mT; turquoise arrows in the numerical simulation). In the unreinforced sample **(B)** mean twist is $\psi =145^{\circ }\pm 12^{\circ }$ (where $180^{\circ }$ would indicate a complete reversal of the PM about *z*) and mean bend is $\theta =6^{\circ }\pm 5^{\circ }$. In the reinforced sample **(D)** mean twist is reduced to $\psi =49^{\circ }\pm 5^{\circ }$ and, due to preservation of magnetic energy, mean bend increases to $\theta =40^{\circ }\pm 7^{\circ }$. The instability which is observed in real world experiments **(B)** clearly appears in the unreinforced state in the FE Simulation **(A)**. This twist is shown more strikingly from the superior view in the upper inset of **(A)**. In the reinforced numerical model **(C)** twisting is completely eradicated by a double helix of PLA at a fiber angle of $85^{\circ }$. This is shown from the superior view in the lower inset. Videos of these deformations are available in the Supplementary Material.

To demonstrate minimization of unstable twisting in the FE simulation we show Figures 7A,C. Optimal fiber reinforcement would represent PLA $\left(E_{PLA}\;=\;2\text{.}4\;\text{GPa}\right)$ at a filament diameter of d= 130 μm. In practice, the thinnest filament we managed to extrude for this study was d= 400 μm; suboptimal by a factor of three but sufficiently close to the optimal diameter to demonstrate our concept. The simulated result for the unreinforced tentacle (Figure 7A and Supplementary Video 1) clearly illustrates the issue we are trying to eradicate. In a twist free solution we would expect to see twist: $\psi =0^{\circ }$ and all rotation being concentrated into bend: θ. This is what we see for the reinforced specimen with $\theta =68^{\circ }$. The unreinforced specimen however, taking the inverse solution from the rotation matrix, we see a large twist angle, $\psi =161^{\circ }$, and the attendant reduction in bending, $\theta =10^{\circ }$.

### 5.2 Fabrication

To verify the findings from the FE simulation, we fabricated a design of the same dimensions. Figure 6 outlines the full fabrication process. Helices were fabricated from white PLA (Material 1,613, Ultimaker, Netherlands). Filament was extruded to a fiber of diameter 0.4 mm (±0.02 mm). Cylinders featuring the desired helical groove were 3D printed (RS-F2-GPGR-04, Formlabs, United States) to host the fibers, which were wound around the forms and secured before being subjected to a heat cycle peaking at 60°C for 30 min. This process embedded the helical shape into the filaments allowing them to retain the helix design upon removal from the form. This was repeated for both left and right-handed helices with a helix angle of $\theta_{H}=85^{\circ }$. As depicted in Figure 6B, the lower diameter, clockwise helix was secured in a 65 mm × 4.5 mm cylindrical mold. Cylindrical inserts were also secured inside the helix to create cavities at predefined desired angles for the PMs to be retrofitted. Silicone (Ecoflex00-30, Smooth-On Inc., United States) was mixed with green pigment (Silc Pig, Smooth-On Inc., United States) and degassed in a high vacuum mixer (ARV-310, THINKYMIXER, Japan) at 1,400 rpm, 20.0 kPa for 90 s. The mixture was then injected into the mold and cured at room temperature for 4 h. This cured structure was demolded and set within the outer, anti-clockwize helix, inserts were removed and PMs placed in the resulting cavities. The second molding stage (Figure 6C) then provided 1.5 mm additional diameter of clear EcoFlex00-30 to secure the PMs and the outer helix. An example of the completed design is shown in Figure 6D.

### 5.3 Experimental Result

**FIGURE 8:**
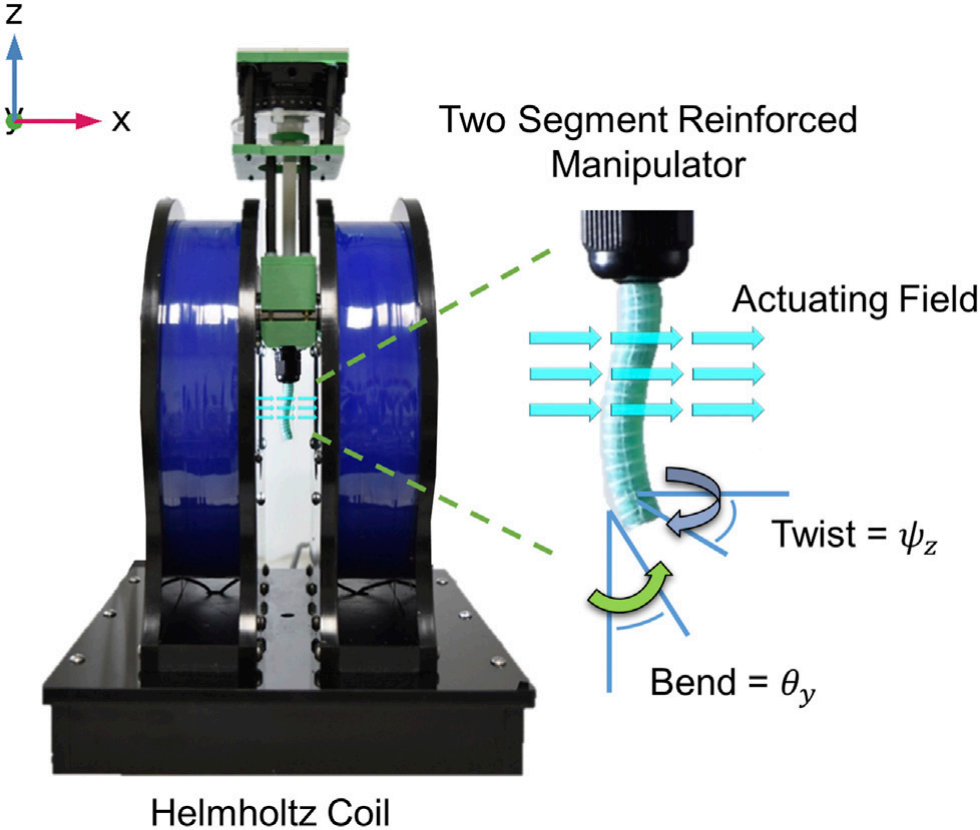
A sample tentacle under actuation in the Helmholtz coil and the two motion primitives being measured: Twist about the local *z* axis (***ψ***) and Bend about the *y* axis (***θ***).

**FIGURE 9:**
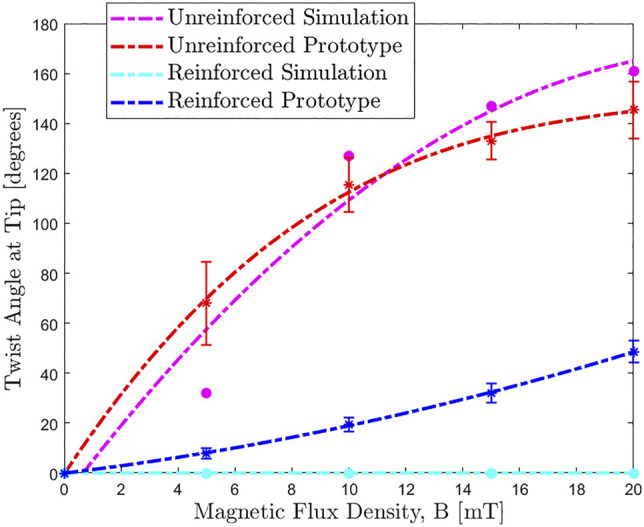
Actuating magnetic flux density (|**B**|) against twist angle at tip (***ψ***) for the unreinforced and reinforced two segment *tentacles* shown in Figure 7. In the reinforced simulation (yellow), twist is completely eradicated for all applied fields. Whilst this success isn’t fully recreated in the reinforced experimental protoype (blue) there is still a significant reduction in unwanted twist across the full spectrum of actuation.

To evaluate the fabricated samples, an actuating field was supplied using a uni-directional Helmholtz coil (DXHC10-200, Dexing Magnet Tech. Co., Ltd., Xiamen, China) as shown in Figure 8. The response to actuation (taken at |B| = 20 mT) of our two segment catheter is shown in Figure 7 (magnetization and actuating field vectors indicated in Figures 7A,C). Angle of twist at the tip of the CM is shown for the range of actuating fields 0 mT ⩽|B|⩽ 20 mT for simulated and experimental results in Figure 9.

Bend angle of the lower PM is derived from the derivative (yellow hatched line) of a fitted second order polynomial (red hatched curve: Polyfit, Matlab version R2018b, the MathWorks, Natick, MA, United States). The angle of twist has been determined by fitting a first order polynomial to imprinted longitudinal lines down the trunk of the segment and measuring the angle *ϕ* between this and the angle of bending at the same z position. This helix angle determines the angle of twist *ψ* via the relationship:

$$tan\left(\phi \right)=\frac{\psi .d}{2.L}.$$ (2)

All experiments were repeated three times under identical conditions. Figures 7A,B are unreinforced and therefore more prone to the twisting instability. In the unreinforced FE model (Figure 7A) we observe a distal twist angle of $\psi =161^{\circ }$ and bend angle of $\theta =10^{\circ }$ at |**B**| = 20 mT (a plot of actuating field against twist angle is shown in Figure 9). The unreinforced experimental sample (Figure 7B) twists through $\psi =145^{\circ }\pm 12^{\circ }$ (as compared to $\psi =49^{\circ }\pm 5^{\circ }$ in the reinforced sample). This twisting results in a loss of magnetic energy in the desired primitive and a consequential reduction in bending at the lower PM, $\theta =6^{\circ }\pm 5^{\circ }$. Figures 7C,D are reinforced with PLA filament $\left(E_{PLA}=2.4\;\text{GPa}\right)$ of diameter d= 400 μm at a fiber angle of $\theta_{H}=85^{\circ }$ and display reduced twist and consequential increase in bend compared to the unreinforced sample. The lower PM shows a bend angle, for the reinforced specimen (Figure 7D), of $\theta =40^{\circ }\pm 7^{\circ }$ as compared to a bend angle of $\theta =68^{\circ }$ in the corresponding FE model (Figure 7C). This discrepancy can be attributed to the continued (but greatly reduced) presence of twist in the experimental prototype as compared to the complete eradication of twist in the idealized FE Model.

## 6 Conclusion and Future Work

In this introductory work, we demonstrate the issue of instability of magnetic CMs under certain configurations of **B** and **m**. We then applied fiber reinforcement, for the first time in magnetic soft robotics, as a means to mitigate this issue. Using our reinforcing at an optimized fiber angle of $85^{\circ }$ we achieve a 78% mean reduction in the twisting primitive and a 67% reduction at |**B**| = 20 mT for our fabricated 40 mm by 6 mm two-segment shape forming catheter. Using our novel design we have significantly reduced the consequences of chaotic input instabilities and created a more robust system which will allow for longer and more varied magnetic catheter profiles.

Limitations in fabrication capabilities have restricted our experimental twist reduction capacity. For practical, large deformation navigations such as gastrointestinal and endovascular interventions we need to exhibit closer reconciliation to numerical results. As such, we hope to develop an automated, miniaturized, and therefore more robust and versatile fabrication technique for variable reinforcement of magnetic catheters. Additionally a more exhaustive optimization procedure for the geometric characteristics of the reinforcement is desirable. In order for the simulation to more closely recreate reality we would also incorporate the unavoidable low magnitude, out of plane input errors in the actuating field which always occur in the real world and also simulate the interaction between multiple embedded permanent magnets. Both small but present contributions to simulation error.

These developments will allow us to move beyond this preliminary phase of study into more thorough and expansive experimental demonstrations. This should offer closer agreement to simulated outcomes and a greater reduction in unwanted twisting which will start to open up the possibility of practical, real-world navigation. From this initial platform, we can look forward to producing multiple-segment catheters capable of stable open-loop navigation through trajectories taken from pre-operative patient images. With this contribution we have taken a step toward fulfilling the potential of magnetic shape forming tentacles for navigation through specific anatomical constraints in a safe and stable manner.

## Data Availability

The original contributions presented in the study are included in the article/Supplementary Material, further inquiries can be directed to the corresponding author.
